# Combined open calcaneocuboid, naviculocuneiform and subtalar dislocation: A case report and literature review

**DOI:** 10.37796/2211-8039.1022

**Published:** 2020-06-05

**Authors:** Khai Phang Wong, Zhi Hao Tang, Gek Meng Tan

**Affiliations:** Department of Orthopaedic Surgery, Khoo Teck Puat Hospital, Singapore

**Keywords:** chopart joint, fracture-dislocation, lateral column, medial column, midfoot injury

## Abstract

Open dislocations of the midfoot and subtalar joints are extremely rare injuries. Understanding the anatomy of these joints and the various injury patterns is imperative to obtain stable concentric reduction and provide good functional outcome. We present a report of a 26- year old male who was involved in a road traffic accident and sustained open dislocations of the calcaneocuboid, naviculocuneiform and subtalar joints. He initially underwent external fixation in view of the severe soft tissue injury. After improvement of the soft tissue condition, he underwent K-wiring of the calcaneocuboid joint, buttress plating of the talonaviculocuneiform joint, peroneal tendon reconstruction using hamstring allograft and defect coverage with a free anterolateral thigh flap. With appropriate rehabilitation protocols, patient recovered well and was allowed to weight bear as tolerated by 10 weeks. His wounds healed completely by 4 months. We report this case considering the rarity of the combined calcaneocuboid, naviculocuneiform and subtalar dislocations which were successfully managed.

## 1. Introduction

Dislocations of the midfoot and subtalar joints are extremely rare injuries, with only a handful of cases being reported in the literature [1e4]. Knowledge of the anatomy and biomechanics of these joints as well as understanding of the injury pattern is essential for the treating surgeon to provide the patient a good functional outcome. We present a rare case of an open dislocation of the calcaneocuboid, naviculocuneiform and subtalar joints, and discuss the diagnosis and management of such an injury.

## 2. Case Report

**Fig. 1:**
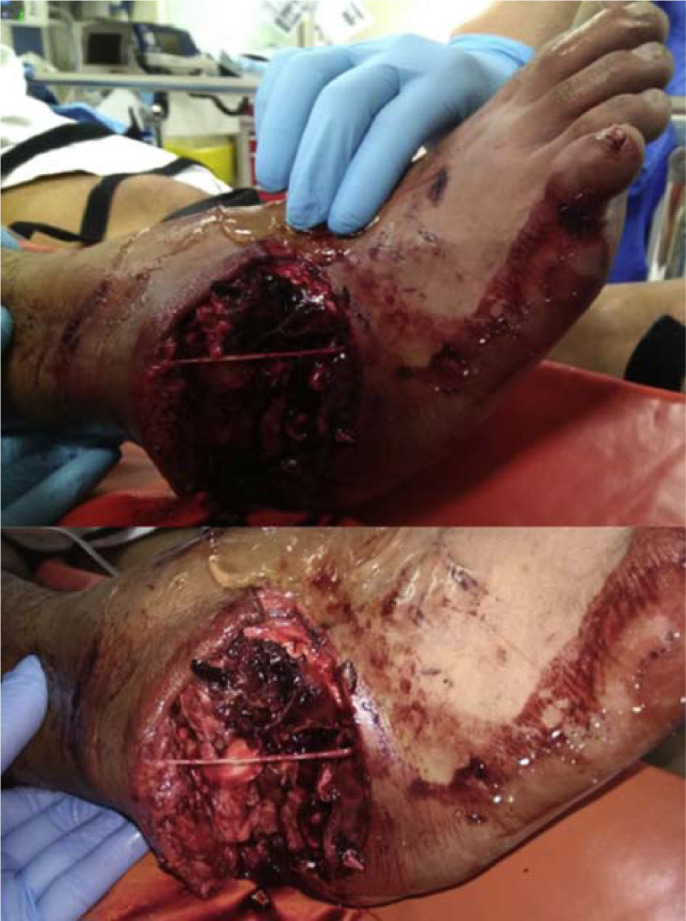
Clinical photograph of the lateral ankle wound with gross ankle deformity.

A 26-year-old otherwise healthy man presented to the Emergency Department following a road traffic accident. He was a motorcyclist who was hit by a taxi. He complained of severe right ankle and foot pain. Clinical examination revealed an open wound over the lateral aspect of his ankle with gross ankle deformity noted (Fig. 1). Posterior tibial pulse was strong but dorsalis pedis pulse was not felt. The foot appeared well-perfused with a capillary refill time of less than two seconds. There was reduced sensation over the lateral aspect of his foot.

**Fig. 2:**
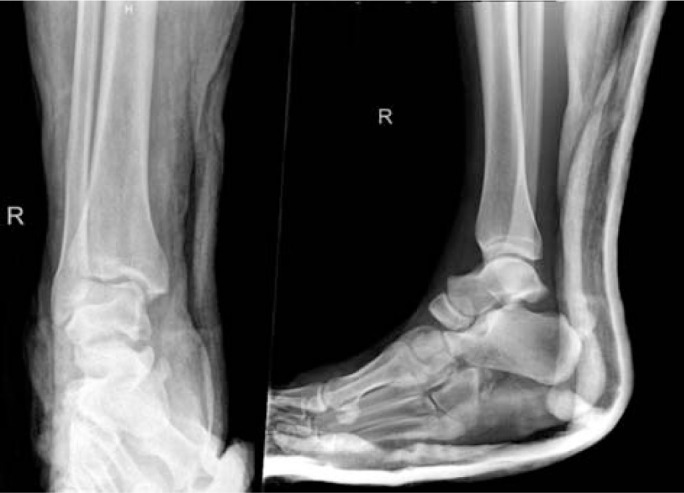
Anteroposterior and lateral ankle radiographs showing dislocations of the subtalar, calcaneocuboid and naviculocuneiform joints.

In view of the absent dorsalis pedis pulse, attempted reduction was performed in the emergency department prior to obtaining radiographs. Post-reduction radiographs revealed dislocations of the calcaneocuboid, naviculocuneiform and subtalar joints (Fig. 2). A computed tomography (CT) angiogram revealed a traumatic occlusion of the dorsalis pedis artery with a patent posterior tibial artery. Vascular consult was made and decision was for conservative management in view of good single vessel runoff.

**Fig. 3:**
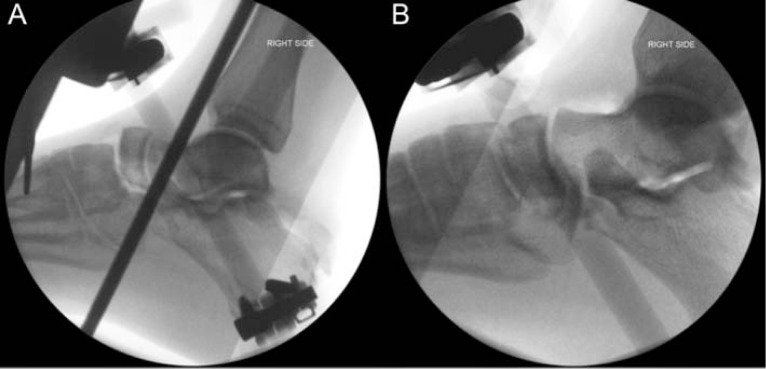
Intraoperative fluoroscopic images showing pre (A) and post (B) reduction of the naviculocuneiform joints. Dorsiflexion and eversion of the foot provided maximum stability.

The patient underwent emergent open reduction and external fixation with Shanz pins placed in the tibia, calcaneus and first metatarsal. The foot was maintained in dorsiflexion and eversion, a position of maximum stability (Fig. 3). Intraoperatively, the peroneal tendons, the lateral ankle ligaments and the talocalcaneal ligaments were found to be cut.

**Fig. 4:**
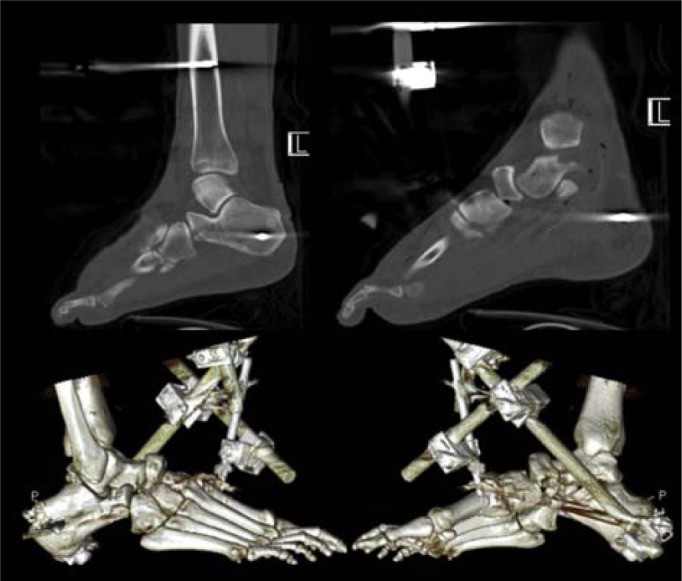
Computed tomography scan showing the unstable calcaneocuboid and naviculocuneiform joints, with a partially extruded navicular bone. Fractures of the cuboid and anterior calcaneal process were also defined.

A CT scan was performed post-external fixation to evaluate for congruency of reduction and to better characterize the injuries. The calcaneocuboid, naviculocuneiform and subtalar joints were still identified to be unstable, with partial extrusion of the navicular (Fig. 4). In addition, fractures of the cuboid as well as the anterior process of the calcaneus were identified.

**Fig. 5:**
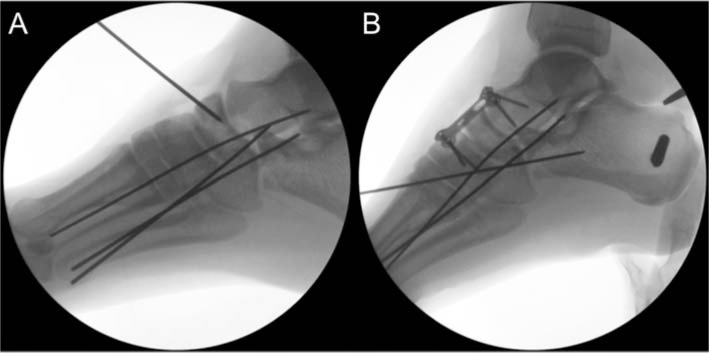
1.6 mm K wires were inserted to reduce the navicular bone and to hold the talonavicular, naviculocuneiform and calcaneocuboid joints in position (A). The navicular bone was then held with a buttress plate spanning the talonaviculocuneiform joints (B).

**Fig. 6:**
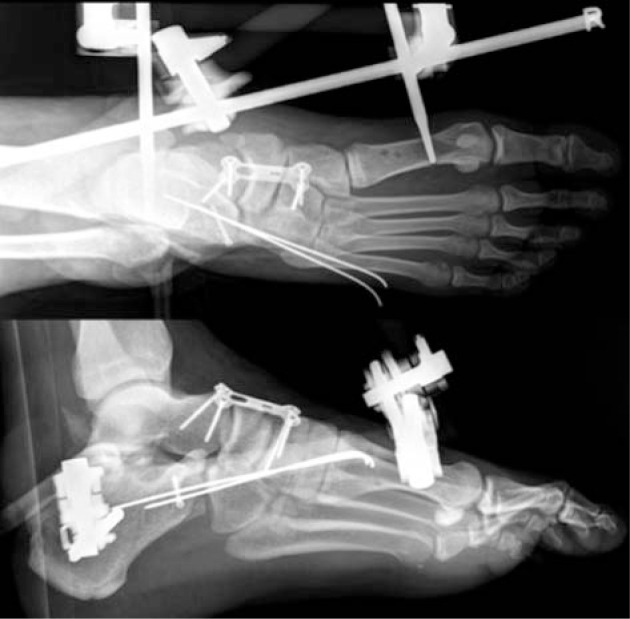
Immediate post-operative radiographs showing good reduction of the calcaneocuboid and naviculocuneiform joints. The external fixator was left in-situ for additional stability.

The patient underwent definitive fixation at a delayed setting after improvement in the soft tissue condition. The subtalar joint was reduced by removing the interposed soft tissues. The talonaviculocuneiform joint was reduced and held with a dorsal medial buttress plate, and the calcaneocuboid joint was held with 1.6 mm Kirschner wires (Figs. 5 and 6). The calcaneal anterior process fracture was also reduced and fixed with a 2.5 mm screw. The external fixator was left in-situ for additional stability. The peroneal tendons were reconstructed using hamstring allograft and the defect was covered with an anterolateral thigh flap.

**Fig. 7:**
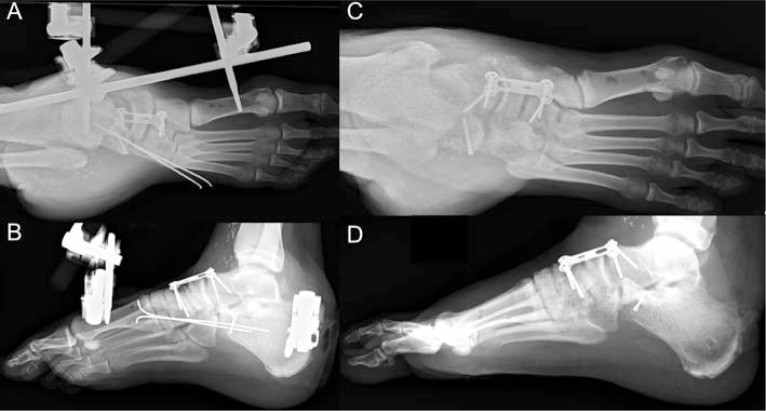
Radiographs at 6 weeks (A, B) and 3 months (C, D) post-operatively showing cell-reduced calcaneocuboid and naviculocuneiform joints. The external fixator was removed after 6 weeks and the patient was allowed to bear weight at 10 weeks.

**Fig. 8:**
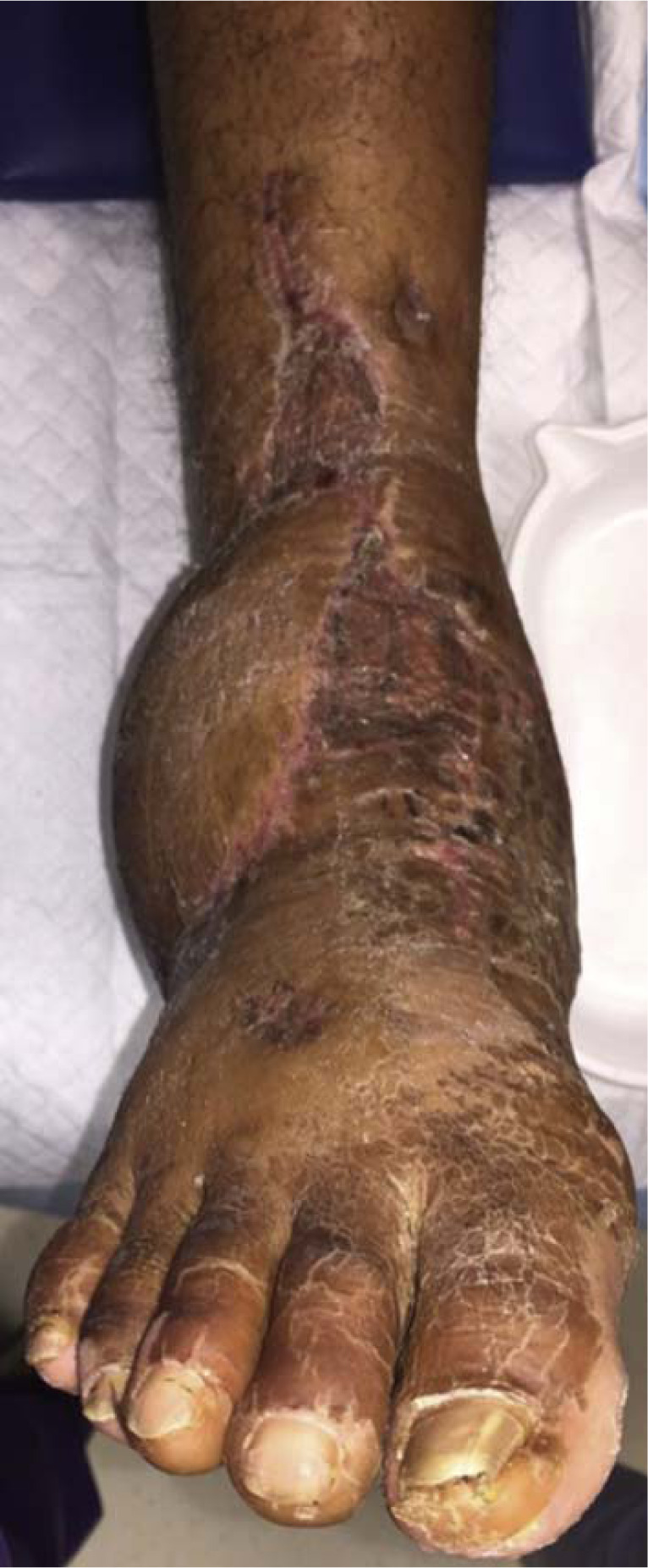
Clinical photograph at 4 months showing well-healed wounds.

The patient recovered well post-operatively. The external fixator and K wires were removed at 6 weeks after adequate healing has occurred. At 10 weeks, there was no more pain or tenderness at the surgical site, and he was allowed weight bearing as tolerated. Serial radiographs revealed no implant loosening with the naviculocuneiform, calcaneocuboid and subtalar joints well-reduced (Fig. 7). At 4 months, his wounds were completely healed (Fig. 8).

## 3. Discussion

Midfoot dislocations are extremely rare injuries. Main and Jowett reported 71 cases of midtarsal injuries with only 10 involving midfoot dislocations [5]. Isolated calcaneocuboid and naviculocuneiform joints have been reported separately [6–9], but there have only been a handful of combined injuries reported in the literature [1–4], with concomitant subtalar dislocations making this case an even rarer entity.

It is important to recognize the anatomy and biomechanics of the midfoot and subtalar joints and the injury patterns associated with such dislocations so as to obtain stable concentric reduction and provide good functional outcomes.

### 3.1. Anatomy of the naviculocuneiform, calcaneocuboid and subtalar joints

Articulation of the navicular bone with the three cuneiform bones consists of three facets with a common synovial capsule [10]. The surrounding interosseous ligaments and tendons restrict movement at the naviculocuneiform joint, providing stability for the transverse arch and the medial longitudinal column.

The calcaneocuboid articulation has its stability conferred by the firm ligamentous attachment to adjacent bones, its relationship to the peroneus longus and brevis tendons, its rigid fibrous capsule and its saddle-shaped joint surface [11]. The stable calcaneocuboid joint is important in maintaining integrity of the transverse arch and the lateral longitudinal column.

For midfoot dislocations, anatomical realignment of the axes and columns is crucial because a reduction of column length or a shift in foot axis will significantly alter gait biomechanics and increase the risk of midfoot arthritis in the future [12].

The subtalar joint consists of the anterior, middle and posterior facets, through which the talus articulates with the calcaneus, permitting foot inversion and eversion. The joint is stabilized by its inherent bony configuration and the supporting soft tissue structures. The talocalcaneal interosseous ligament within the sinus tarsi and capsular structures around each facet, strengthened medially by the superficial deltoid ligament and laterally by the calcaneofibular ligament, constitute the main ligamentous support [11]. Studies have shown that the calcaneofibular, superficial deltoid and talocalcaneal ligaments must all tear for medial or lateral dislocations to occur, with the spring ligament remaining relatively uninjured [13]. This is true of our present case, where the lateral ligaments and the talocalcaneal ligaments were found to be cut, with the superficial deltoid ligament not explored.

### 3.2. Mechanism of injury

Midfoot dislocations are usually sustained after high energy trauma, such as road traffic accidents as shown in our case report. Such injuries usually involve both osseous and ligamentous components, involve both medial and lateral columns and are almost always associated with significant soft tissue injury.

Kollmannsberger et al. [7] postulated that in isolated calcaneocuboid dislocations, the mechanism is an inverting swivel of the forefoot distal to the midtarsal joint about an axis through the navicular. In such injuries, the inherent stability of the joint usually preserves the reduction after a successful closed reduction. However, in our present case, the associated medial column injury and ligamentous disruption precluded a stable reduction and the patient had to be brought to the operating theatre for surgical stabilization. In addition, CT scan showed a partially extruded navicular, suggesting perinavicular instability which might further complicate attempts at closed reduction.

Main and Jowett classified their cases based on the direction of deforming forces [5]. Their cases were thought to be as a result of plantarly directed forces on the midfoot, resulting in a plantar dislocation of the midfoot in relation to the hindfoot. In our report, the forces are postulated to be the same as the midfoot is also dislocated plantarly one articulation distal to the talonavicular joint.

Subtalar dislocations are usually classified by the direction the foot takes in relation to the talus [14,15]. Medial subtalar dislocations are more commonly reported. These occur when there is forced eversion of the plantarflexed foot, with the sustentaculum tali acting as a fulcrum for the posterior talar body. Lateral subtalar dislocations are comparatively rarer and they usually occur after forceful eversion of the plantarflexed foot. The anterolateral talus acts as a fulcrum about which the anterior process of the calcaneus pivots. In our present case, it is likely that a continuation of these forces leads to the calcaneocuboid and naviculocuneiform dislocations, producing a “peritalar dislocation” [11].

### 3.3. Clinical evaluation

Early accurate diagnosis and appropriate treatment is important to prevent long term complications and functional impairment. A high index of suspicion is important in such high energy injuries and radiographs should be obtained early. In our case, radiographs were only obtained after the initial reduction due to the fact that this occurred in a trauma activation setting where other scans were being prioritized first and also the concern about vascular compromise. We acknowledge this as a limitation and do not recommend routinely attempting reduction without prior radiographs identifying the exact injuries. Signs and symptoms of compartment syndrome should also be monitored for, in view of the severe soft tissue compromise.

Initial radiographic evaluation should consist of anteroposterior, oblique and lateral views of the foot. Ebraheim et al recommended routine medial oblique views to best visualize the calcaneocuboid joint line [16]. Further imaging such as a CT scan is useful to pick up associated fractures not readily identified on initial radiographs [17,18]. In our case report, the CT scan showed additional cuboid and calcaneal fractures not previously evident.

### 3.4. Treatment

Although it is generally agreed that such cases of midfoot dislocations require reduction, there is a general lack of consensus in the literature as to which is the recommended method of fixation. Previous cases have been treated with K wires [3], plate fixation [2] and closed reduction under fluoroscopy [1]. Unlike isolated calcaneocuboid dislocations in which closed reduction may be successful due to the congruency of the joint preserving the reduction [7], combined dislocations are usually more unstable and are more likely to require open reduction. This is also seen in our case report where the calcaneocuboid and naviculocuneiform joints remained unstable even after external fixation.

Similarly, closed reduction is successful in most cases of isolated subtalar dislocations [19]. However, the complex nature of this injury possibly precluded successful closed reduction. Other possible obstacles to closed reduction of medial dislocations include button-holing of the talar head through the extensor retinaculum or the talonavicular capsule, interposition of the deep peroneal neurovascular bundle or peroneal tendons, and impaction of the lateral navicular into the medial talar head [11]. The authors acknowledge the initial inadequate reduction of the subtalar joint due to the complex injury pattern, which was rectified with open reduction during the definitive surgery after improvement of the soft tissue condition.

Our principles of managing such a complex injury consists of initial soft tissue management, interval definitive fixation to restore medial and lateral column length, definitive soft tissue stabilization and eventual skin coverage. In accordance to the principles of management of open fractures, we elected to apply an external fixator in the emergent setting in view of the large open wound and the severe soft tissue trauma.

Plate fixation has been shown to provide rigid fixation without further articular damage as compared to transarticular screws [20]. Plate fixation has also been shown to be stiffer and has a high load to failure. For this reason, we elected for plate fixation of the naviculocuneiform and talonavicular joints. Bridge plating was used as a spanning construct across the naviculocuneiform and talonavicular joints, providing a buttress to counter dorsal subluxation of the navicular. Anatomical reconstruction of the naviculocuneiform joint is of primary importance due to the role of the medial column in maintaining the arch of the foot and the stronger ligaments permitting it less motion compared to the calcaneocuboid joint. In view of the large lateral-sided wound, the calcaneocuboid joint was held with K wires to minimize hardware complications. The small calcaneal anterior process fracture was fixed with a 2.5 mm screw.

Redfern and Myerson previously described a treatment algorithm for combined tears of the peroneus longus and brevis, based on a validated protocol [21]. Surgical options depend on the presence of excursion of the proximal muscle. If there is no excursion of the proximal muscle, a tendon transfer procedure is recommended, typically with the flexor digitorum longus tendon. If excursion is present, an allograft tendon reconstruction is the procedure of choice. This can either be a one-stage or two-stage procedure, depending on presence of tendon scarring. If minimal or no scarring is present, a one-stage procedure can be performed. However, in the presence of tendon scarring, the surgery can be staged, with the first stage involving using a silicone rod to reestablish a synovial cavity and the definitive procedure performed 6 weeks later [22]. In our present case, the peroneal tendons were reconstructed using gracilis tendon allograft, and the torn anterior capsule and lateral ligaments addressed with an open Brostrom repair.

## 4. Conclusion

Open dislocations of both the calcaneocuboid and naviculocuneiform joints are rare injuries. Understanding the anatomy of the midfoot joints is of utmost importance in treating such injuries. Principles of management include initial soft tissue management, interval definitive fixation and soft tissue reconstruction and repair to restore medial and lateral column length for good clinical and functional outcome.
